# The cyanobacterial neurotoxin β-N-methylamino-l-alanine (BMAA) targets the olfactory bulb region

**DOI:** 10.1007/s00204-020-02775-6

**Published:** 2020-05-20

**Authors:** Paula Pierozan, Elena Piras, Eva Brittebo, Oskar Karlsson

**Affiliations:** 1 Science for Life Laboratory, Department of Environmental Science, Stockholm University, 114 18 Stockholm, Sweden; 2 Department of Pharmaceutical Biosciences, Uppsala University, Box 591, 751 24 Uppsala, Sweden

**Keywords:** BMAA, Olfactory bulb, mGluR, NMDAR, Neurodegenerative diseases, ALS/PDC, Intranasal, Air pollution

## Abstract

Olfactory dysfunction is implicated in neurodegenerative disorders and typically manifests years before other symptoms. The cyanobacterial neurotoxin β-N-methylamino-l-alanine (BMAA) is suggested as a risk factor for neurodegenerative disease. Detection of BMAA in air filters has increased the concern that aerosolization may lead to human BMAA exposure through the air. The aim of this study was to determine if BMAA targets the olfactory system. Autoradiographic imaging showed a distinct localization of radioactivity in the right olfactory mucosa and bulb following a unilateral intranasal instillation of ^3^H-BMAA (0.018 µg) in mice, demonstrating a direct transfer of BMAA via the olfactory pathways to the brain circumventing the blood–brain barrier, which was confirmed by liquid scintillation. Treatment of mouse primary olfactory bulb cells with 100 µM BMAA for 24 h caused a disruption of the neurite network, formation of dendritic varicosities and reduced cell viability. The NMDA receptor antagonist MK-801 and the metabotropic glutamate receptor antagonist MCPG protected against the BMAA-induced alterations, demonstrating the importance of glutamatergic mechanisms. The ionotropic non-NMDA receptor antagonist CNQX prevented the BMAA-induced decrease of cell viability in mixed cultures containing both neuronal and glial cells, but not in cultures with neurons only, suggesting a role of neuron–glial interactions and glial AMPA receptors in the BMAA-induced toxicity. The results show that the olfactory region may be a target for BMAA following inhalation exposure. Further studies on the relations between environmental olfactory toxicants and neurodegenerative disorders are warranted.

## Introduction

Olfactory dysfunction is a common feature of neurodegenerative disorders such as Parkinson disease (PD), parkinsonism–dementia complex (PDC), dementia with Lewy bodies and Alzheimer’s disease (AD), and it has also been demonstrated in amyotrophic lateral sclerosis (ALS) (Attems et al. 2014; Zou et al. 2016). Olfactory impairment appears early in the neurodegenerative process (Marin et al. 2018) and the olfactory bulb pathology is suggested to progress to other central brain regions (Fullard et al. 2017).

Olfaction has an essential role in behavior and memory. Olfactory dysfunction is commonly linked to pathological deposition of proteins, such as α-synuclein, hyperphosphorylated tau and neurofilament proteins in the olfactory system (Attems et al. 2014; Wesson et al. 2010). This accumulation can induce a complex cascade of molecular events including oxidative stress, neuroinflammation and cytosolic disruption of cellular processes that lead to cell death (Pearce et al. 1995). In PD, the typical α-synuclein aggregates are observed very early in the olfactory bulb (Braak et al. 2003) and affects 95% of all cases (Sabbagh et al. 2009). Since the olfactory bulb is not protected by the blood–brain barrier (Oberdorster et al. 2004), this brain region is continuously exposed to airborne pollutants (Franco et al. 2007). Airborne environmental contaminants as well as pharmaceuticals locally administrated on the nasal mucus may be directly transferred to the olfactory bulb along the axons of the olfactory neurons, as their dendrites project into the nasal mucus and their axons into the olfactory bulb (Bergstrom et al. 2002; Eriksson et al. 1999). Pathological alterations induced by airborne environmental toxicants may contribute to the initiation of protein aggregation in the olfactory bulb, which in turn triggers the spread of the pathology within the brain (Attems et al. 2014). Clarifying whether the changes in the olfactory bulb could be a starting point of pathological processes is therefore important to understand the etiology of neurodegenerative diseases.

The cyanobacterial neurotoxin β-N-methylamino-l-alanine (BMAA) has been implicated as a possible risk factor for Guamanian ALS/PDC (Caller et al. 2018). The discovery that ubiquitous cyanobacteria are capable of producing BMAA in freshwater and marine water bodies has highlighted BMAA as a global environmental hazard (Cox et al. 2018). Since aerosolization of cyanobacteria and cyanotoxins is known to occur, inhalation through wind or marine recreational sports may provide a route of exposure to BMAA (Caller et al. 2018; Cheng et al. 2007). Caller and coworkers reported that a population residing close to water bodies that frequently experience dense cyanobacterial blooms has a 10–25 times higher ALS incidence compared to the normal population (Caller et al. 2009). This is in line with a recent study demonstrating an increased risk of ALS in subjects residing within 100 m from water bodies (Maria et al. 2020). Detection of BMAA in air filters has further increased the concern that aerosolization may lead to human BMAA exposure through the air (Banack et al. 2015). Cyanotoxins in airborne desert dust has also been suggested to be responsible for the high rates of ALS among Persian Gulf War veterans (Cox et al. 2009).

BMAA is a mixed glutamate receptor agonist that can act via multiple mechanisms including induction of oxidative stress, inhibition of melatonin synthesis (important neuroprotective hormone), disruption of neuronal stem cell proliferation and differentiation (Lobner 2009; Pierozan et al. 2018a; Pierozan and Karlsson 2019), as well as triggering neuronal cell death (Karlsson et al. 2011; Lobner 2009). Studies also show that BMAA may be associated with, or incorporated into, proteins (Dunlop et al. 2013; Karlsson et al 2009a; Karlsson et al 2015b) and induce protein misfolding, accumulation of protein aggregates, intracellular fibril formation, increased protein ubiquitination, α-synuclein deposition and apoptosis, supporting a role for BMAA in the development of neurodegeneration (Karlsson et al. 2015a, 2012, 2014; Neonatal exposure to BMAA can induce significant behavioral changes in adult rats including long-term cognitive impairment (Karlsson et al. 2009b, 2009c, 2011). Other studies also report that BMAA-exposed rats demonstrate neuropathology similar to neurodegenerative disease and behaviors indicating hyposmia and lack of odor identification and/or discrimination (Scott and Downing 2017a, 2017b), typical symptoms of early stages of PD and AD (Alves et al. 2014; Kranick and Duda 2008).

The aim of this study was to determine if BMAA is transferred to the olfactory region after intranasal administration in mice and to study the acute effects of BMAA on neuronal morphology and cell viability in primary mouse olfactory bulb cultures.

## Method

### Materials

β-N-Methylamino-l-alanine [methyl-^3^H] was obtained from Biotrend GmbH, Cologne, Germany. The specific radioactivity was 85 Ci⁄mmol and the radiochemical purity was 99%. β-N-Methylamino-l-alanine hydrochloride (≥ 97% purity), paraformaldehyde, 4′,6-diamidino-2-phenylindole dihydrochloride (DAPI), Triton X-100, 3-(4,5-dimethyl-2-thiazolyl)-2,5-diphenyl-2H-tetrazolium bromide (MTT), dimethyl sulfoxide (DMSO), laminin, poly-D-lysin and Ara-C were obtained from Sigma-Aldrich (St Louis, MO, USA). Bovine serum, penicillin–streptomycin, Dulbecco’s phosphate-buffered saline (PBS), Dulbecco’s modified Eagle's medium (DMEM), Hanks' balanced salt solution (HBSS), neurobasal medium, trypsin solution (0.05%), glutamine, B27 and olfactory marker protein (OMP) anti-rabbit were obtained from Gibco (Invitrogen, Paisley, UK). The secondary antibodies Alexa-Fluor 555 goat anti-mouse IgG, 488 goat anti-rabbit IgG were obtained from Molecular Probes (Invitrogen, Paisley, UK). The NMDAR antagonist MK-801, the non-NMDAR ionotropic antagonist 6-cyano-7-nitroquinoxaline-2,3-dione (CNQX) and mGluR antagonist (S)-α-Methyl-4-carboxyphenylglycine (MCPG) were obtained from Tocris Bioscience (Bristol, UK). The antibody β III-tubulin anti-mouse was obtained from Abcam (Cambridge, UK).

### Animal and housing

Adult male mice and time-mated pregnant C57BL/6 J mice were purchased from Taconic (Ejby, Denmark) and housed in Makrolon cages (59 × 38 × 20 cm) containing wood-chip bedding and nesting material. The animals were maintained on standard pellet food (R36 Labfor; Lantmännen, Kimstad, Sweden) and water ad libitum, in a temperature and humidity-controlled environment on a 12-h light/dark cycle. All animal experiments were performed according to protocols approved by the Uppsala Animal Ethical Committee and in accordance with the Swedish Legislation on Animal Experimentation (Animal Welfare Act SFS1998:56) and the European Union Directive on the Protection of Animals Used for Scientific Purposes (2010/63/EU).

### Intranasal administration of ^3^H-BMAA in mice

Twenty-five adult male C57BL/6 J mice were anesthetized with an intraperitoneal injection of a combination of ketamine and xylazine (100 mg and 10 mg/kg body weight, respectively). A trace dose of ^3^H-BMAA (10 µCi, 0.018 µg) dissolved in HBSS was administered unilaterally to the right nostril (5 µL) using polyethylene tubes (PE-10) attached to a micropipette. The mice were then placed on their right sides on a heating pad (38 °C) until awake. The animals were euthanized by CO_2_ after 30 min, 1, 3, 5 and 24 h, and the olfactory bulbs, posterior cortex, anterior cortex and the remaining parts of the cerebrum were collected. The samples were weighed and dissolved in 1 ml tissue solubilizer (Solune-350; Perkin-Elmer, Boston, MA, USA) at 50 °C overnight. After that, 10 ml scintillation cocktail (Hionic Fluor; Perkin-Elmer, Boston, MA, USA) was added and the radioactivity was measured in a liquid scintillation analyzer (Tri-Carb 1900CA; Packard Instruments Company, Downers Grove, IL, USA). To study intranasal uptake and brain distribution in detail, autoradiographic imaging was conducted on five animals as previously described (Karlsson et al. 2009a). Briefly, the heads were embedded in aqueous carboxymethyl cellulose and frozen in a cyclohexane dry-ice bath. Series of transversal tissue sections (20 µm) were collected on tape at various levels, freeze-dried and processed for autoradiography using X-ray film (Hyperfilm-3H; Amersham Biosciences, Uppsala, Sweden). The exposure of the film was performed at − 20 °C.

### Effects of BMAA on primary mouse olfactory bulb cells

Pregnant C57BL/6 J mice were euthanized by decapitation (Jerneren et al. 2015; Pierozan et al. 2017) at embryonic day 18 and the olfactory bulbs were dissected under microscope from the embryos. The olfactory bulbs were transferred into HBSS without any Ca^2+^ or Mg^2^ and cut into small pieces and rinsed three times in HBSS before incubation in a 0.025% trypsin–EDTA solution for 30 min at 37 ℃. The enzymatic digestion was stopped by addition of fetal bovine serum (FBS). Mechanical dissociation was performed using Pasteur pipettes. Cells were centrifuged at 200* g* for 5 min, resuspended in neurobasal medium containing 2 mM glutamine, 100 U/ml penicillin/streptomycin, B27 supplement and 10 µM Ara-C to isolate neurons for the neuronal cultures. For mixed cell cultures, DMEM/F12 medium supplemented with 10% FBS and 100 U/mL penicillin/streptomycin was used. Viable cells were counted using an automatic cell counter (Countess^®^ II, Invitrogen, Paisley, UK) and plated at a density of 100,000 cells/cm^2^ in 96-well plates coated with poly-D-lysine substrate and laminin. Cells were maintained in a 37 ℃ humidified 95% air-5% CO_2_ atmosphere (Panasonic, MCO-170AICUVH-PE, Osaka, JP). The culture medium was replaced the day after seeding and then every third day. After 7 days in vitro, cells were exposed for 24 h to 50 or 100 µM (neuronal cultures) and 250 or 500 µM BMAA (mixed glial and neuronal cultures) dissolved in culture medium. The concentrations used were based on a pilot study. In the experiments designed to study signaling mechanisms triggered by BMAA, both mixed and neuronal cultures were preincubated with 100 µM MK-801, 25 µM CNQX, or 50 µM MCPG for 30 min and then co-exposed with BMAA for 24 h (Pierozan et al. 2018b). All experiments were repeated three times.

### Cell viability assay

Cell viability was measured by the MTT assay as previously described (Pierozan et al. 2018c). The formazan product generated during the incubation with 0.5 mg MTT was solubilized in dimethyl sulfoxide (DMSO) and measured at 490 nm using a POLARstar OTIMA microplate reader (BMG LABTECH, Offenburg, Germany).

### Immunocytochemistry

Immunocytochemistry was conducted as previously described (Pierozan et al. 2018a). In short, mouse olfactory bulb neuronal cells were plated in 96-well plates at a density of 40,000/cm^2^ and treated with BMAA (50–500 µM) for 24 h. Cells were then fixed with 4% paraformaldehyde for 30 min and permeabilized with 0.1% Triton X-100 in PBS for 5 min at room temperature. After blocking with 1% bovine serum albumin, the fixed cells were incubated overnight with anti-β III-tubulin (1:200) and OMP (1:500) antibodies at room temperature, followed by PBS washes and incubation with specific secondary antibodies conjugated with Alexa 488 (sheep anti-rabbit, 1:1000) or Alexa 555 (sheep anti-mouse,1:1000) for 1 h. In all immunostaining, negative controls reactions were performed by omitting the primary antibody. No reactivity was observed when the primary antibody was excluded. The cell nuclei were stained with DAPI (0.25 mg/mL).

### Morphometric analysis by high-content imaging 

Images of the primary mouse olfactory bulb neuronal cells were collected with a 10X objective in an ImageXpress Micro XLS Widefield High-Content Analysis System (Molecular Devices, Sunnyvale CA, USA), and the images were analyzed with the SoftMax Pro Software after digital acquisition (Molecular Devices, Sunnyvale CA, USA) using the MetaXpress neurite outgrowth application module based on β III-tubulin staining. Quantitative analysis of the cultured cells was conducted in nine microscopic fields per well and six wells per group.

### Statistics

Cell culture results are presented as mean values ± standard deviations (S.D.). Data from the experiments were analyzed statistically by one-way ANOVA (control vs treated) or two-way ANOVA (co-treatment with glutamate antagonists) followed by Tukey–Kramer test when the *F* test was significant using the GraphPad Prism 7 software. *P* < 0.05 was considered significant.

## Results

### Transfer of ^3^H-BMAA to the mouse olfactory bulb following intranasal administration

**Fig. 1:**
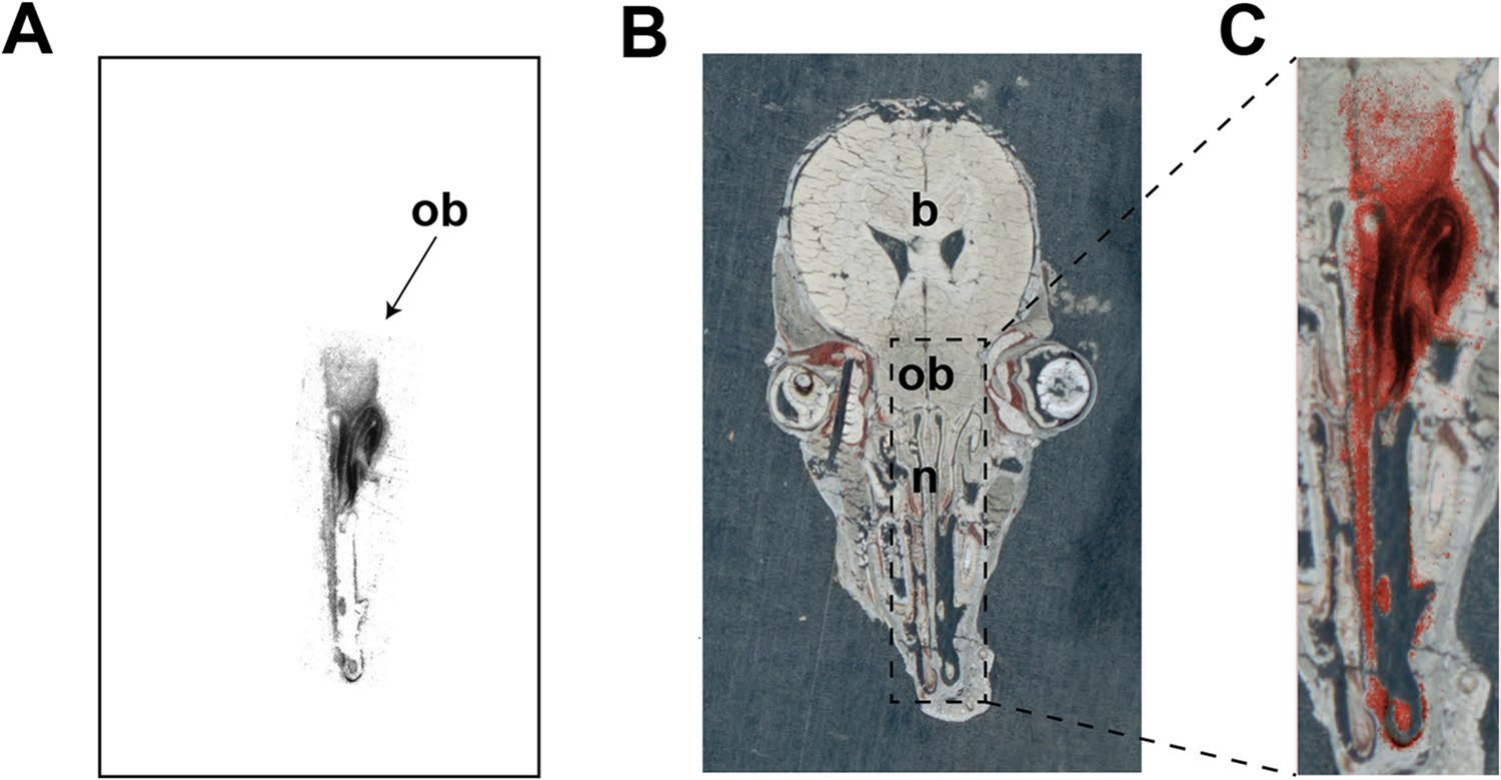
Transfer of ^3^H-BMAA to the mouse olfactory bulb following a unilateral intranasal administration. **(a)** A representative autoradiogram showing a high and distinct localization of radioactivity in the right nasal olfactory mucosa, the right axonal nerve layer and peripheral layer of the olfactory bulb (black areas) 24 h after unilateral intranasal administration of ^3^H-BMAA (10 µCi, 0.018 µg) in the right nostril of a mouse. No radioactivity can be seen in the left nostril and olfactory region showing that there is no transfer of radioactivity from the treated right nostril. **(b)** The corresponding freeze-dried transversal tissue section of the mouse head. **(c)** The enlarged overlay image shows the localization of radioactivity in the right olfactory region highlighted in red. Nasal region (n), olfactory bulbs (ob) and brain (b) (color figure online)

The autoradiographic imaging of freeze-dried tissue sections revealed a distinct transfer and selective localization of radioactivity in the right nostril, nasal olfactory mucosa and olfactory bulb after a unilateral ^3^H-BMAA (10 µCi, 0.018 µg) administration in the right nostril (Fig. 1a, b and c).

As shown in Table 1, the liquid scintillation study confirmed the autoradiographic data and demonstrated markedly higher levels of radioactivity in the right olfactory bulb compared with the left olfactory bulb, at all examined post-injection times (30 min, 1, 2, 5, 24 h). Radioactivity was detected also in other brain regions at levels approximately one order of magnitude lower than in the right olfactory bulb (Table 1).

**Table 1:** Brain tissue levels of radioactivity following a unilateral administration of ^3^H-BMAA^1^ in the right nostril of mice

Brain region	^3^H-BMAA DPM/mg tissue (wet weight)^2^				
	30 min	1 h	2 h	5 h	24 h
Right olfactory bulb	2785 ± 1204	2296 ± 757	7860 ± 1905	7188 ± 1119	1287 ± 365
Left olfactory bulb	353 ± 63	540 ± 105	560 ± 66	469 ± 48	268 ± 45
Anterior cortex	159 ± 14	238 ± 21	250 ± 19	259 ± 48	202 ± 28
Posterior cortex	140 ± 19	198 ± 17	217 ± 21	203 ± 52	201 ± 29
Remaining cerebrum	168 ± 9	210 ± 25	188 ± 30	218 ± 42	187 ± 24

^1^10 µCi corresponding to 0.018 µg

^2^Each value represents mean ± SEM (*n* = 3–4 animals)

### Glutamate-mediated effects of BMAA on mouse olfactory bulb cell viability

**Fig. 2:**
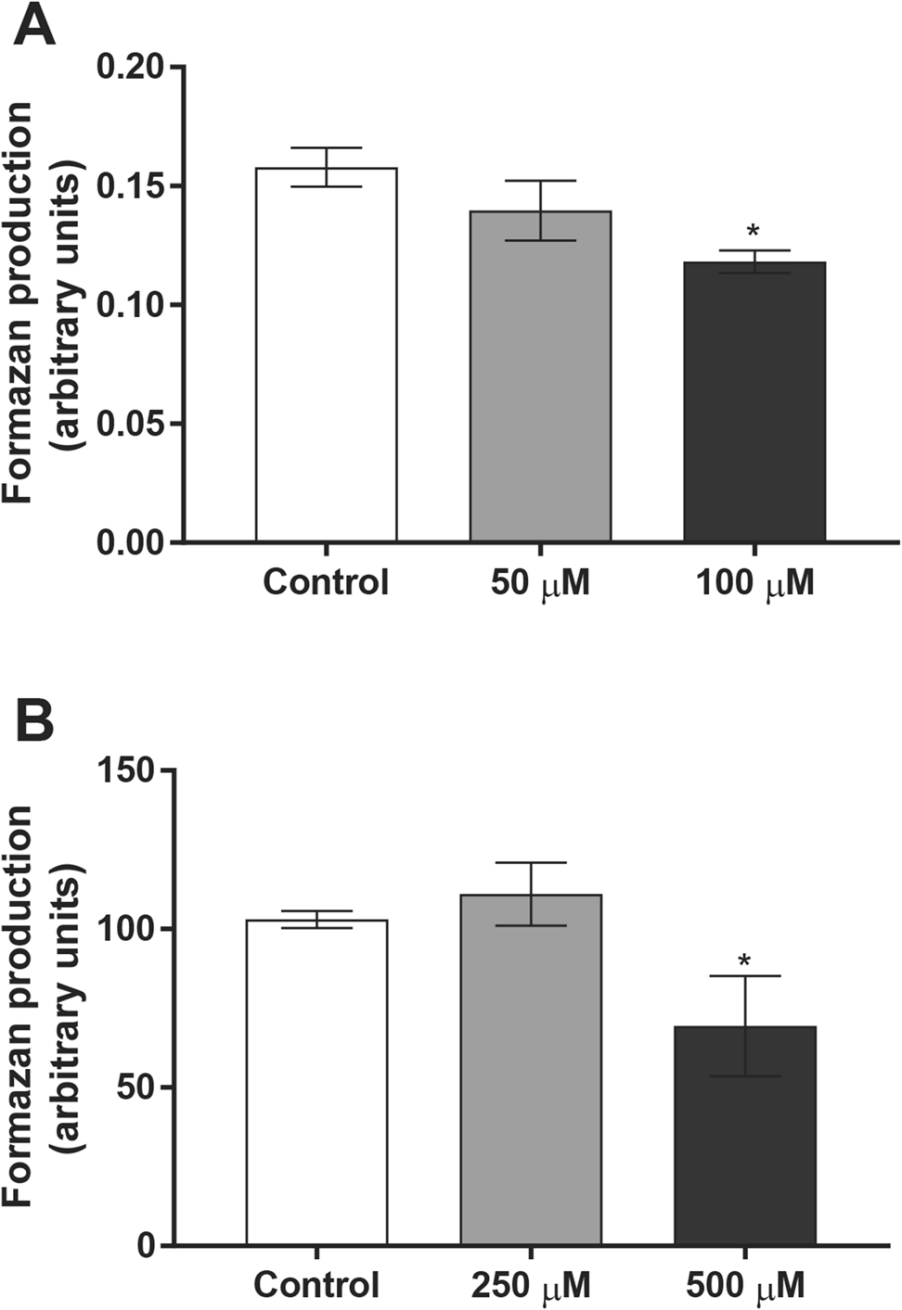
BMAA-induced toxicity in primary mouse olfactory bulb cells. Primary mouse olfactory bulb neurons **(a)** or olfactory bulb mixed neuronal/glial cultures **(b)** were treated with 50 µM to 500 µM BMAA, or cell medium for 24 h. Cell viability was measured by MTT assay. Data are expressed as mean ± S.D. of three independent experiments, based on three separate culture preparations. Each experiment includes four replicates. Statistical differences (one-way ANOVA followed by Tukey–Kramer test) are indicated as follow: *p < 0.05 compared with control cultures grown in cell medium only

The effects of BMAA on the viability of olfactory bulb neurons and mixed olfactory bulb cultures were examined following 24 h exposure. Neuronal cell cultures were exposed to 50 or 100 µM BMAA and mixed olfactory bulb cultures were exposed to 250 or 500 µM BMAA. Neuronal olfactory bulb cultures demonstrated a 25% decrease in cell viability at exposure to 100 µM BMAA compared with the control (Fig. 2a), while in the mixed cell cultures a 31% cell viability decrease was observed at 500 µM BMAA (Fig. 2b). Lower BMAA concentrations had no effects on cell viability after 24 h exposure in any of the cell culture types (Fig. 2b).

**Fig. 3:**
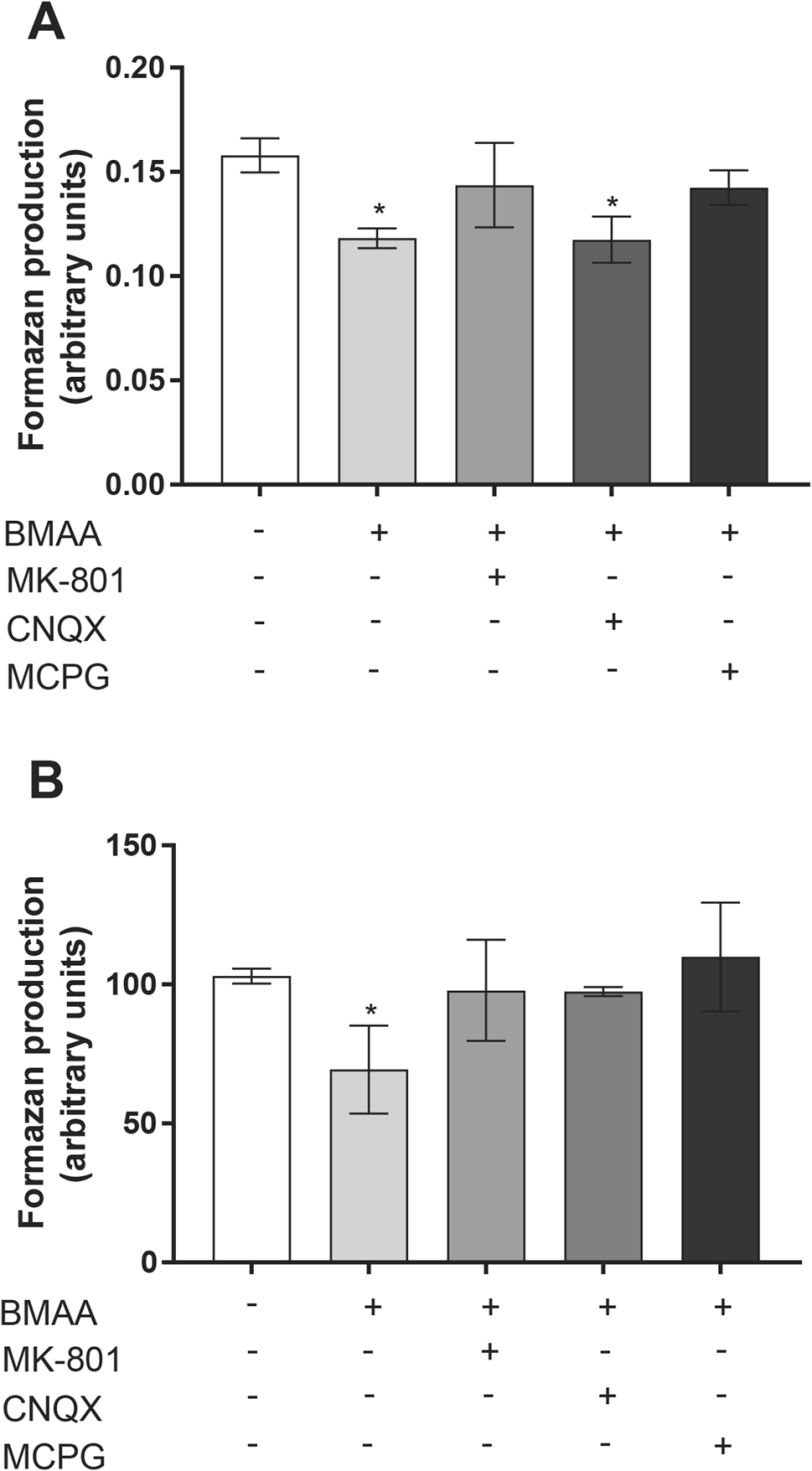
BMAA-induced toxicity via activation of glutamatergic receptors. Primary mouse olfactory bulb neuronal **(a)** or mixed neuronal/glial olfactory bulb **(b)** cultures were preincubated for 30 min with the NMDAR antagonist MK-801 (100 µM), the non-NMDAR antagonist CNQX (25 µM), the mGluR antagonist MCPG (50 µM), or cell medium only, followed by co-treatment with 100 µM (neurons) or 500 µM (mixed cultures) BMAA for 24 h. Cell viability was measured by MTT assay. Data are reported as mean ± S.D. of three independent experiments, based on three separate olfactory bulb culture preparations. Each experiment included four replicates. Statistical differences (two-way ANOVA followed by the Tukey–Kramer test) are indicated as follows: *p < 0.05 compared with control group grown in cell medium only

Since BMAA can act as a glutamate agonist (Lobner 2009), the ability of glutamate antagonists to prevent the BMAA-induced effects was examined. The NMDAR antagonist MK-801 and the mGluR antagonist MCPG were able to partially prevent the reduced cell viability caused by BMAA both in olfactory bulb neurons and mixed olfactory bulb cultures. Interestingly, the non-NMDAR ionotropic antagonist CNQX failed to prevent these effects in olfactory bulb neurons, but partially prevented the BMAA-induced effects in the mixed olfactory bulb cultures (Fig. 3 and b).

### Reduction of neurite outgrowth by BMAA in olfactory bulb neurons

**Fig. 4:**
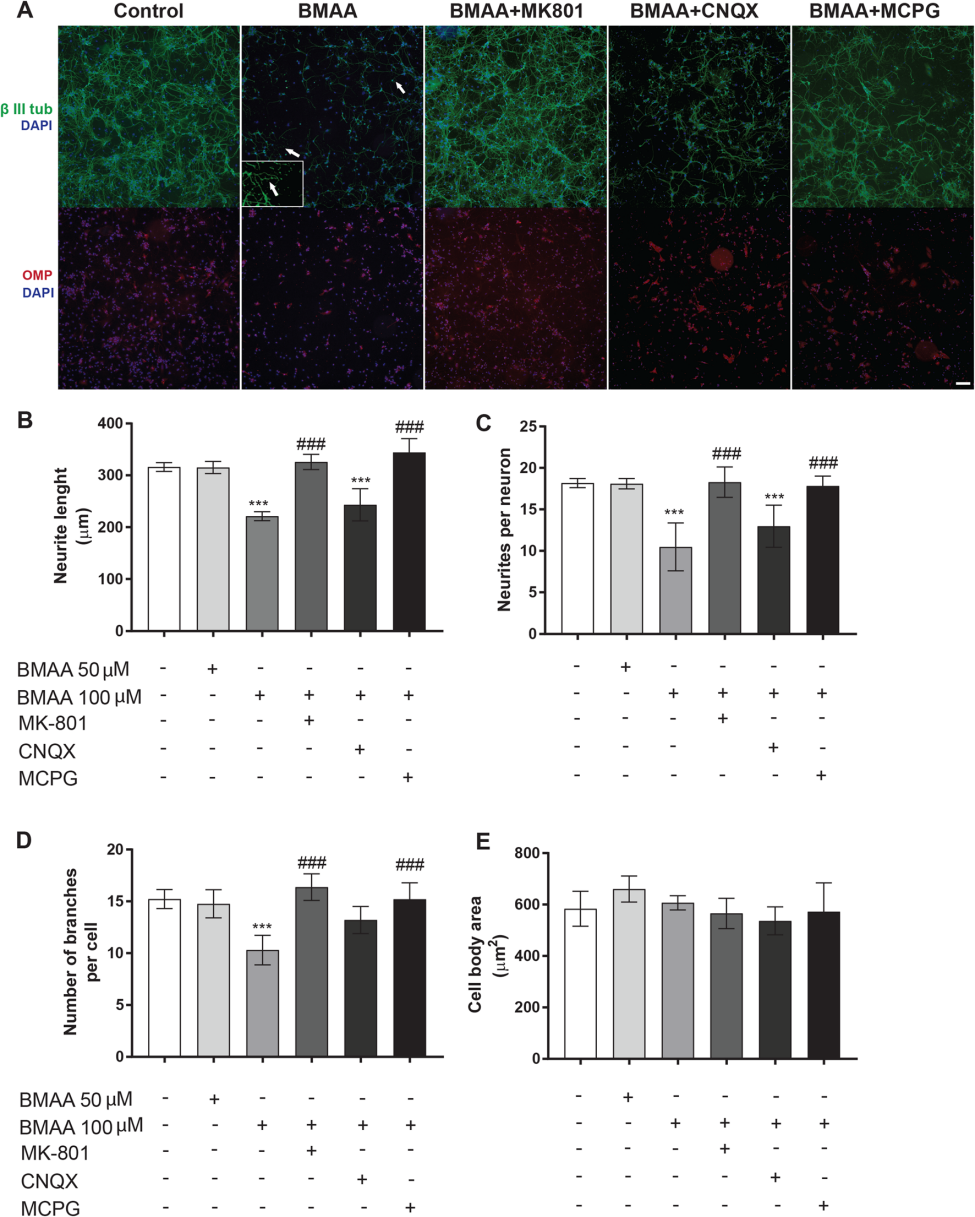
BMAA-induced morphometric alterations in primary mouse olfactory bulb neurons. **(a)** Representative images of cells exposed to BMAA for 24 h or pre-treated and co-exposed to the NMDAR antagonist MK-801, mGluR antagonist MCPG or non-NMDAR ionotropic antagonist CNQX. The primary mouse cell cultures were immunostained with anti-β III-tubulin (green), anti-OMP (red) and DAPI (blue). Dendritic varicosities are present in olfactory neurons treated with 100 µM BMAA (arrows, enlarged in the inserted frame). Images were captured with an ImageXpress Micro XLS Widefield High-Content Analysis System (Molecular Devices, Sunnyvale CA, USA). The morphometric analysis of the cells was conducted with the SoftMax Pro Software and included: neurite length **(b)**, processes per cell **(c)**, branches per cell **(d)** and cell body area **(e)**. Data are reported as mean ± S.D. of three independent experiments, based on three separate olfactory bulb neuron culture preparations. Each experiment included six replicates. Statistical differences (two-way ANOVA followed by the Tukey–Kramer test) are indicated as follows: ***P < 0.001 compared with control group grown in cell medium only, and ^###^*p* < 0.001 compared with the cultures treated with 100 µM BMAA. Scale bar = 50 µm (color figure online)

To examine if BMAA can perturb the neuronal process outgrowth, morphological characterization of neurites was conducted in olfactory bulb neurons cultured during 8 days and treated with 50 or 100 µM BMAA for 24 h. Immunocytochemical staining with 488-labeled anti-β III tubulin and 555-labeled anti-OMP was conducted before images were captured and analyzed with the High-Content Analysis System. All cell cultures were positive for OMP (Fig. 4a). Control olfactory neurons demonstrated complex neurite networks with long processes. Exposure to 50 µM BMAA induced no significant morphological alterations, while neurons treated with 100 µM BMAA displayed a disrupted neurite network, with formation of dendritic varicosities (Fig. 4a, arrow). The morphometric analysis revealed a reduced neurite outgrowth (Fig. 4b), decreased number of neurites per neuron (Fig. 4c) and number of branches per neurons (Fig. 4d), without any effect on the cell body area (Fig. 4e). The pre-/co-treatment of olfactory bulb neurons with the NMDAR antagonist MK-801 or the mGluR antagonist MCPG completely prevented the BMAA-induced effects. In contrast, the non-NMDAR ionotropic antagonist CNQX failed to protect against the BMAA-induced effects on neuronal morphology (Fig. 4b, c, d and e).

## Discussion

The cyanobacterial neurotoxin BMAA has been associated with various neurodegenerative disorders and inhalation through wind or marine recreational sports is a potential route of exposure to BMAA (Caller et al. 2018; Cheng et al. 2007; Scott et al. 2018). The present study demonstrated a transfer of radioactivity along the olfactory pathways to the olfactory bulb after intranasal administration of ^3^H-labeled BMAA in mice. The bipolar olfactory neurons have dendrites projecting into the nasal mucus and axons projecting into the olfactory bulb. Taken together, this suggests that there is direct transfer of BMAA via the olfactory pathways to the brain, circumventing the blood–brain barrier. BMAA was further shown to disrupt the neurite network and viability of cultured primary olfactory bulb neuronal cells via activation of both NMDA and metabotropic glutamatergic receptors, suggesting that the olfactory bulb may be a sensitive target tissue.

The major neuronal elements of the olfactory bulb include the afferent olfactory neurons, the output mitral and tufted (M/T) cells and the intrinsic periglomerular and granule cells (Trombley and Shepherd 1993). Aggregates of proteins such as α-synuclein have been detected in olfactory neurons (Niu et al. 2018), which can trigger complex cascades of oxidative damage, neuroinflammation and excitotoxicity leading to cell death (Pearce et al. 1995). Although cell loss in the olfactory bulb has not been directly investigated in neurodegenerative disease brains, the demonstrated olfactory bulb volume reduction clearly indicates ongoing cell death in this brain region (Li et al. 2016; Rey et al. 2018).

Glutamate released from M/T cells mediates dendrodendritic transmission at synapses with granule cells through ionotropic receptor subtypes (Aroniadou-Anderjaska et al. 1999). The granule cell excitation triggers GABA release from the dendrites, which inhibits M/T cells via GABA receptors (Schoppa and Urban 2003). During overactivation of ionotropic glutamate receptors, rapid increases in intracellular calcium can occur, which is a key signal for neuronal injury (Dong et al. 2009). The NMDAR antagonist MK-801 protected against the BMAA-induced neurite alterations and decreased viability in primary olfactory neuronal cell cultures, demonstrating the role of ionotropic glutamate receptor in the mechanism of action. The non-NMDAR ionotropic antagonist CNQX failed to prevent these effects in olfactory neuronal cell cultures. This is in line with a previous report showing that olfactory bulb neurons are sensitive to NMDA-induced toxicity, but relatively insensitive to AMPA-induced injury (Farso et al. 2006).

Our results also revealed a key role of metabotropic glutamate receptors in the BMAA-induced decrease of neurite outgrowth and viability in primary olfactory bulb neurons. M/T cells express high levels of mGluR1, while granule cells express high levels of mGluR5 (Heinbockel et al. 2007). mGluR1 and mGluR5 are coupled to the inositol triphosphate/calcium pathway and activation of these receptors stimulates an increase of calcium release from internal stores in olfactory bulb cells, which can trigger delayed cell death processes (Carlson et al. 2000; Geiling and Schild 1996). It is important to note that cultured primary olfactory bulb neurons were more susceptible to BMAA exposure than mixed primary olfactory bulb cultures that also contain glial cells. This is in agreement with studies showing that cultured rat glial cells from the olfactory system, the olfactory ensheathing cells, are less sensitive to BMAA (Chiu et al. 2013). In addition, current evidence shows that glial cells may influence the neuronal survival and activity through direct cell-to-cell contacts and via a variety of soluble factors (Schmalenbach and Muller 1993).

The olfactory glial cells share some of the properties of both astrocytes and Schwann cells, but appear to have advantages over other glial cells regarding CNS repair (Barnett and Riddell 2004). They can decrease neuronal apoptosis, reduce glial scarring, phagocytosis of axonal debris and produce a number of trophic factors that help axonal recovery (Au and Roskams 2003; Barnett and Riddell 2004; Delaviz et al. 2008). This indicates a role of glial AMPA receptors in the BMAA-induced effects as the non-NMDAR ionotropic antagonist CNQX prevented the BMAA-induced cytotoxicity in the mixed olfactory bulb cultures, but could not modulate the BMAA-induced toxicity in the primary olfactory bulb neurons.

Early stages of many neurodegenerative diseases and age-related degeneration are characterized by neurite damage and compromised synaptic function that precede neuronal cell death (Fiala et al. 2002). Evidence points out the role of excitotoxicity in dendritic injury (Hasbani et al. 1998, 2001; Hasel et al. 2015). Sustained elevation of intracellular calcium caused by glutamate inhibits the dendrite outgrowth and causes the formation of focal swellings (varicosities) along the length of the dendritic arbor (Hasbani et al. 2001). The presence of dendritic varicosities has been documented under several pathological conditions (Hsu and Buzsaki 1993; Luebke et al. 2010), and one possible explanation is that alterations in dendritic morphology resulted from glutamate receptor-mediated calcium entry that triggers the destruction of neuronal elements responsible for dendritic structure (Hasbani et al. 2001). This is in accordance with the present findings that the NMDAR and the mGluR antagonists prevented decreased neurite outgrowth and varicosities formation caused by BMAA in neuronal olfactory bulb cultures. Since the BMAA concentration that decreased neurite outgrowth also caused reduced cell viability, we cannot exclude that the decreased neurite outgrowth could also be due to cell death processes. However, as the mechanisms of cell death include distinct sets of biochemical and morphological changes such as alteration of cell volume (Bortner and Cidlowski 2002; Elmore 2007), the unaffected cell body area in BMAA-treated cultured olfactory bulb neurons suggests a specific calcium-mediated inhibition of neurite outgrowth.

In conclusion, the present findings show that the cyanobacterial neurotoxin BMAA is directly transferred into the olfactory bulb through the olfactory pathways, circumventing the blood–brain barrier in mice and that BMAA can induce cytotoxicity in primary olfactory neurons via glutaminergic receptors. This suggest that the olfactory region may be a target for BMAA-induced toxicity following inhalation exposure. Given the link between pathologic process in the olfactory system and development of neurodegenerative disease, these findings could also be relevant for understanding the underlying mechanisms of olfactory dysfunction and the association of the neurotoxin BMAA with neurodegenerative disorders. More studies of the BMAA-induced effects on olfactory bulb cells are warranted, as pathology in this brain area possibly can progress to other central brain structures.
